# Medical considerations in the care of transgender and gender diverse patients with eating disorders

**DOI:** 10.1186/s40337-022-00699-3

**Published:** 2022-11-21

**Authors:** Megan C. Riddle, Joshua D. Safer

**Affiliations:** 1 Eating Recovery Center, 1231 116Th Ave NE, Bellevue, WA 98004 USA; 2 Department of Psychiatry and Behavioral Sciences, University of Washington, 1959 NE Pacific Street, Box 356560, Seattle, WA 98195-6560 USA; 3 Mount Sinai Center for Transgender Medicine and Surgery, 275 7Th Ave 12Th Floor, New York, NY 10001 USA

**Keywords:** Transgender, Gender diverse, Eating disorder, Gender affirming care, Medical complications

## Abstract

Transgender and gender diverse (TGD) individuals are at increased risk for the development of eating disorders, but very little has been published with regards to the unique aspects of their medical care in eating disorder treatment. Providing gender affirming care is a critical component of culturally competent eating disorder treatment. This includes knowledge of gender affirming medical and surgical interventions and how such interventions may be impacted by eating disordered behaviors, as well as the role of such interventions in eating disorder treatment and recovery. TGD individuals face barriers to care, and one of these can be provider knowledge. By better understanding these needs, clinicians can actively reduce barriers and ensure TGD individuals are provided with appropriate care. This review synthesizes the available literature regarding the medical care of TGD patients and those of patients with eating disorders and highlights areas for further research.

## Plain English summary

Transgender and gender diverse (TGD) people are at increased risk for developing eating disorders, but very little is known about their unique medical needs while in eating disorder treatment. TGD refers to individuals whose sex reported at birth does not align with their gender identity. This review examines the existing literature on TGD medical care and integrates this with the eating disorder literature. Improved knowledge of the medical needs to TGD individuals can help decrease barriers to care. This review aims to better understand the medical needs of TGD individuals in eating disorder treatment and highlights areas for further research.

## Introduction

While traditionally young, cisgender women were thought to be the population with the highest prevalence of eating disorders, increasingly, data suggest that transgender and gender diverse (TGD) individuals may be at even greater risk [1–3]. Transgender is an adjective that describes when someone’s gender identity is incongruent with their sex recorded at birth (Table 1). Gender identity refers to a person's inward sense of their sex. Individuals may identify as either male or female, or somewhere outside the binary, such as gender diverse, non-binary, genderqueer, pangender, agender or genderfluid [4]. Gender expression refers to the outward presentation of an individual’s gender, and may include things like clothes, hairstyle, behavior, and interests. No term perfectly encapsulates the gender diverse population; this article uses the terms transgender and gender diverse (TGD) to describe these individuals.

**Table 1:** Definition of terms

Transgender	When someone’s gender identity is incongruent with their sex recorded at birth
Cisgender	When someone’s gender identity is congruent with their sex recorded at birth
Gender identity	A person's inward sense of their sex
Gender diverse	A term to describe when one’s gender identity does not fit solely within the “male” or “female” gender binary
Sex recorded at birth	The sex recorded on the birth certificate
Gender expression	The outward presentation of an individual’s gender, which may include things like clothes, hairstyle, behavior, and interests
Gender affirming hormone treatment (GAHT)	Medication that replaces the sex hormone profile with one that corresponds with a person’s gender identity in order to achieve physical characteristics that are more aligned with an individual’s gender identity
Gender affirming surgery	Surgical interventions that seek to align physical characteristics with an individual’s gender identity
Social affirmation	Changes to an individual’s name, pronouns, clothing and appearance to align with their gender identity
Legal affirmation	Changes made to legal documents such as birth certificates, school documents and passports with updated names and gender markers

Emerging data show TGD individuals are at particularly increased risk for eating disordered behaviors. Diemer and colleagues’ survey of nearly 300,000 college students found that 15.82% of transgender individuals reported a diagnosis of an eating disorder in the past year, compared to 1.85% of cisgender heterosexual women [2]. Similarly, of youth presenting at a clinic for gender-affirming care, 15% had increased rates of eating disordered behaviors and 63% had attempted to alter their weight for gender-affirming purposes [5]. In a large community sample of TGD individuals in the US, 13.8% of gender diverse individuals, 10.6% of transgender men, and 8.1% of transgender women reported being told they had an eating disorder by a mental health or medical provider [6, 7]. Of the patients entering care at large United States-based ED treatment center, 6% of adult patients identified as TGD on admission [8], notably higher than the prevalence of transgender individuals in the general US population of 0.6% [9].

Several factors have been proposed for the increased risk of disordered eating in TGD individuals. Eating disordered behaviors have been postulated to serve to alter physical characteristics in ways that align with an individual’s gender identity [1, 10, 11], and body dissatisfaction has been shown in previous research to be a risk factor for the development of disordered eating in this population [12]. TGD individuals are also subject to significant stress due to being a minoritized population; the minority stress hypothesis posits that TGD individuals face ongoing chronic stressors due to being a member of a historically excluded group, leading to increased rates of physical and mental health issues, including eating disorders. Consistent with this, Watson and colleagues found higher rates of eating disordered behaviors among TGD youth who had experienced higher rates of harassment and discrimination [13]. TGD individuals also face increased rates of food insecurity, which may contribute to disordered eating [14, 15]. Limited research has examined factors that are protective against the development of disordered eating. These protective factors include a sense of connection with family and at school, as well as other forms of social supports [13].

As a field, we have become increasingly aware of the risk of eating disorders in the TGD population. However, knowledge of TGD health tends to be lacking among providers, including those providing eating disorder treatment. The National Transgender Discrimination Survey found 50% of participants reported needing to teach their providers about trans health [16]. While TGD individuals have been shown to access healthcare at lower rates than cisgender individuals, having a healthcare provider knowledgeable about trans health was the most important predictor of accessing care [17]. Previous research looking specifically at patients’ experiences in eating disorder treatment reported patients felt their gender identity was at times ignored by the primary care provider or they were misgendered [18]; in another study, 62% of participants specifically noted eating disorder treatment providers lacked training in TGD health care [19]. When providers have inadequate knowledge of TGD health care this can create further barriers to TGD patients receiving adequate care and may hinder their eating disorder treatment [18].

While the research suggests TGD individuals are often aware of their gender identity prior to entering adolescence, many do not present for gender affirming care until later in adolescence or early adulthood [20, 21]. As with eating disorders, family involvement can have a significant impact on when an individual presents for gender-affirming care, with those presenting at a later age often having less support around their gender identity [22]. Research also suggests that those presenting to care later have increased rates of mental health problems [23]. Given peak eating disorder onset is also adolescence and early adulthood, this may necessitate a care team helping an individual recover from their eating disorder while simultaneously supporting their gender journey and providing gender-affirming care. Previous work had expressed concerns that transgender identity might be transient and significant barriers were in place to decreases access to gender-affirming care. Recent studies have reported rates of retransitioning (i.e. returning to their sex recorded at birth) to be very low [24] and those who do retransition most frequently do so at least in part due to external factors, such as pressure from family members or non-affirming work or school environments [25]. As such, it is clinically appropriate to support these patients in their exploration and affirmation of their gender by providing gender-affirming care.

In this article, we seek to summarize medical management of TGD individuals with an emphasis on where this overlaps and interacts with eating disorder treatment and its complications. Despite growing interest in this area of research with most of the publications produced in the past five years, there remain significant gaps in our knowledge of how best to care for TGD individuals with eating disorders. We also highlight the role of medical providers in determining access to care, and how the intersectional nature of trans identity may impact access to and quality of medical care, as patients may be members of more than one marginalized group. Of note, a full discussion of gender-affirming medical interventions is beyond the scope of this article but we would refer readers to other excellent reviews previously published on this topic [9, 26] as well as guidelines provided by World Professional Association of Transgender Health (WPATH SOC 8) and Endocrine Society [27, 28] (Table 2).

**Table 2:** Recommended resources

World Professional Association of Transgender Health, Standards of Care for the Health of Transgender and Gender Diverse People, Version 8	https://www.wpath.org/
UCSF Transgender Care & Treatment Guidelines	https://transcare.ucsf.edu/guidelines
Endocrine Society Clinical Practice Guidelines	https://www.endocrine.org/clinical-practice-guidelines/gender-dysphoria-gender-incongruence

## Gender affirming care

TGD individuals may pursue gender affirmation in several different domains, including social, legal, medical, and surgical [29]. Social affirmation can include gender affirming changes to an individual’s name, pronouns, clothing and appearance [30]. Individuals may change legal documents such as birth certificates, school documents and passports with updated names and gender markers [29]. Medical and surgical interventions, outlined below, seek to align physical characteristics with the individual’s gender identity. Gender affirming interventions are customized to the patient. All patients should be asked about their name and pronouns on intake and staff are encouraged to share their own pronouns during introductions (Table 3). Care teams should use correct names and pronouns for patients regardless of whether the patient is present, including in the medical record [29]. The exception to this is if the patient requests a different name or pronoun be used with family members; in this case, the patient’s request for privacy regarding their gender identity should be protected. Team members should be encouraged to practice correct pronoun use and those who misgender a patient should be corrected in the moment. During the exam, trauma-informed care is necessary as many patients have experienced trauma, including within the medical system [31]. Language is important in the creation of safety and, in addition to use of correct name and pronoun in the exam, providers are encouraged to use gender neutral language whenever possible [31]. Ideally the clinician uses the terminology the patient uses to refer to parts of their own body. Clinicians should be conscientious about asking questions that are medically necessary and not simply out of curiosity. If a mistake is made with regards to the patient’s gender, pronouns or other terminology, apologize. The environment also plays a role in providing safe spaces. Medical facilities are encouraged to have gender neutral bathrooms. When rooms are shared, it is recommended that patients be placed with roommates of the same gender identity [32]. In addition, all staff need to be trained in gender-affirming care to create a safe care environment [33, 34].

**Table 3:** Gender affirming practice recommendations

Environment of care	Provide gender neutral bathrooms
	Room patients with those of the same gender identity; avoid isolating patients
	Provide private bathrooms on request
	Use the correct name for patients on daily attendance sheets, room doors, etc.
Staff	Provide regular training on gender-affirming care and practices
	Encourage staff to include their own pronouns in introductions to normalize sharing of correct pronouns
	Intervene if staff or other patients are misgendering a patient, regardless of whether the patient is present
	Support the hiring and retention of TGD staff members; inclusivity should extend beyond the patients
Medical record	Provide gender-affirming intake forms that include what pronouns and name the patient uses
	Use a medical record that allows for the inclusion of gender and pronouns
Clinical practice	During the initial assessment, inquire about how individuals would like to be addressed, including their name and pronouns. This may include a conversation about whether a different name or pronouns are to be used with family members. It is important to protect patient’s privacy regarding their gender identity
	Practice using correct pronouns in all practice settings
	Use gender-related language and terminology the patient prefers. This includes the language that may be used to describe their body parts. Use gender neutral language. i.e. breasts versus chest; uterus and ovaries versus internal parts. If specific parts need to be named, consider using “the” rather than “your,” i.e. “the uterus” instead of “your uterus.”
	Only ask questions to which you clinically need to know the answers
	When appropriate, use an anatomy inventory to determine what screening may need to occur
	Do not make assumptions about what gender affirming medical interventions a patient may have had or may want to consider in the future
	Facilitate patient access to gender affirming medical interventions
	If TGD care is not a regular part of your practice, consider practicing appropriate language when the patient is not present
	Consider seeking TGD CME
	Be conscientious of the impact of implicit bias in clinical decision making

## Gender affirming medical interventions

Whether to pursue gender affirming medical interventions and what interventions to pursue is personalized to the individual. Surveys suggest that approximately 50% of transgender individuals may go on to seek gender affirming surgical or hormonal treatments, with higher rates noted in older populations [16, 35]. Studies have shown improvement in mental health symptoms, including disordered eating behaviors, for those TGD patients receiving gender affirming medical interventions [3, 36, 37].

## Hormonal treatments

The model for obtaining hormone treatment has shifted over the years. Previously, being transgender was viewed as a mental health concern requiring a mental health treatment strategy. TGD people seeking hormone therapy would need to start with mental health treatment and “prove” their transgender status [26]. Current treatment standards suggest that adults starting hormone therapy meet the following criteria: persistent/consistent gender identity, capacity to consent to treatment, and that mental or physical health concerns are assessed and included in the discussion of risks and benefits [27, 28]. This shift away from previous language in recommendations which required “reasonable” control of mental illness is notable, as historically gender-affirming care has been denied or delayed due to the presence of other psychiatric diagnoses [38]. However, new guidelines place the focus on reducing the barriers the psychiatric illness may place on accessing and engaging in gender affirming treatment [27]. This has direct implications for eating disorder treatment as it suggests gender affirming care should not wait until the patient is in recovery, but rather be pursued in parallel to their other treatment.

Broadly speaking, the goal of gender affirming hormone treatment (GAHT) is to achieve physical characteristics that are more aligned with an individual’s gender identity. This is done by replacing the sex hormone profile with one that corresponds with a person’s gender identity [28]. Hormone levels are typically targeted to be at the same level as is found in cisgender individuals. For trans masculine individuals, testosterone is administered with the goal of reaching physiological levels of cisgender men (300 to 1000 ng/dL) [26]. For trans feminine individuals, gender affirming hormones aim to suppress the testosterone level from the typical cisgender male range to the typical cisgender female range (less than 50 ng per deciliter) while keeping estradiol from rising above the typical cisgender female range (less than 200 pg per milliliter) [9]. Gender diverse individuals may also pursue GAHT and may seek hormone levels somewhere along the spectrum between cisgender men and cisgender women [39]. Prior to initiation of GAHT both adolescents and adults should be counseled on the potential impact on fertility [28].

Unlike for adults, TGD adolescents require mental health assessment before beginning gender affirming medical therapy. For TGD care, WPATH SOC 8 defines adolescence as the period of time between the start or puberty and the age of legal majority [27]. Prior to Tanner stage 2 of pubertal development, GAHT is not recommended [28]. Once an individual reaches Tanner stage 2, pubertal suppression with Gonadotropin Releasing Hormone (GnRH) analogues, such as leuprolide, can be considered [40]. This treatment can provide additional time to plan treatment while puberty is paused.

For trans masculine individuals on GAHT, changes induced by endogenous testosterone include increased libido, amenorrhea, increased muscle mass, fat redistribution, facial hair growth, clitoromegaly, and voice deepening [9]. Testosterone can be administered via patch, gel, subcutaneous or intramuscular injection or subcutaneous implants. Medical risks include a decrease in HDL and increase in LDL cholesterol as well as the potential for causing polycythemia [26].

For trans feminine individuals, GAHT works to both decrease testosterone levels and increase estrogen levels. This can be achieved through exogenous estrogens administered via oral, transdermal or parenteral routes [26]. While estrogen alone can suppress testosterone, most studies suggest the addition of adjunctive anti-androgen agents, including spironolactone, GnRH agonists, or cyproterone acetate, to achieve desired levels while also limiting doses of exogenous estrogen [26]. Spironolactone is typically titrated to 100 to 300 mg daily [9]. As this is a potassium-sparing diuretic, serum potassium levels should be monitored, and extra consideration taken in those patients who engage in fluid restriction as part of their eating disorder. Cyproterone is given at a dose of 10 mg daily and leuprolide is provided via intramuscular injection, dosing varying with the frequency (3.75–7.50 mg IM of SC monthly or 11.25–22.50 mg every 3 months). Goals of trans feminine hormones include stimulating breast development, redistributing fat to a more gynoid pattern, and decreasing body hair [26]. Physiological changes can be expected within 3 to 12 months of starting hormone treatment. Medical risks include increased risk of venous thromboembolism (VTE) [26]. While concerns have been raised for increased risk for myocardial infarctions and cerebrovascular events compared to cisgender women, no difference has been found when compared to cisgender men or cisgender women on oral contraceptives [26]. Those using spironolactone should be monitored for hyperkalemia [26].

Restrictive eating disorders cause suppression of the hypothalamic pituitary axis. For those recorded female at birth, restriction can result in hypothalamic amenorrhea with reduction in pulsatile GnRH secretion. This results in a decrease in the amplitude of LH-FSH release and a transition back to a prepubertal pattern [41]. For transgender men and gender diverse individuals recorded female at birth, restriction can lead to a gender congruent amenorrhea and a more androgenous figure; weight restoration may result in a rise in endogenous hormone levels and physical changes that are incongruent with gender identity.

A study of cisgender males with anorexia nervosa (AN), showed testosterone level of 129.5 ng/dL versus healthy cisgender male controls at 496 ng/dL [42]. With weight restoration, hormone levels normalize [41]. Thus, for transgender women, restriction may lead to a gender-congruent suppression of endogenous hormones, while weight restoration can lead to a rise in hormones incongruent with their gender identity.

Extensive review of the literature did not produce any articles providing evidence or guidance regarding the administration of gender-affirming hormones for individuals with comorbid eating disorders. Historically, the presence of mental health issues, including eating disorders, could preclude someone from being eligible for GAHT [43]. Waiting for a patient to achieve full recovery from their eating disorder would serve as a significant barrier to gender-affirming care and may result in further harm to the patient by delaying needed treatment. In fact, the initiation of GAHT during eating disorder recovery may help individuals who are underweight tolerate weight restoration as body fat is redistributed in a way more congruent with their gender identity. Patients who are already taking GAHT should be continued on these medications during treatment. For those not already taking GAHT who would like to do so, the care team should support the patient in pursuing this care.

For patients with restrictive eating disorders already taking GAHT, it may be prudent to measure levels more frequently, given the impact of restriction on the HPA axis. In addition, one case report describes persistent hypophosphatemia past the typical duration seen in refeeding syndrome in a trans woman taking spironolactone and estrogen [44]. Further research is needed to create an evidence base for best practices of hormone treatment during eating disorder treatment.

## Surgical interventions

While reports vary significantly, it is thought that up to half of transgender individuals may seek out gender-affirming surgical procedures [9, 35, 45, 46]. For trans masculine individuals, gender-affirming surgeries include chest reconstruction, hysterectomy and oophorectomy, and genital reconstruction surgeries [9]. For trans feminine individuals, surgeries include breast augmentation, facial feminization, and genital reconstruction surgeries [9]. As with all surgeries, gender-affirming surgeries carry with them the risk of complications, which varies based on the procedure performed. For those undergoing genital surgery, this can include complications to wound healing, including infection and tissue ischemia, or impairments in in function, such as difficulties voiding or changes to physical sensation or sexual function [47]. Risks associated with chest reconstruction or breast augmentation include infection, hematoma and need for revision [48]. Gender affirming surgeries are consistently associated with improved quality of life [48, 49]. Previous studies have suggested that gender affirming surgeries are associated with better mental health and limited data suggest this may be associated with a decrease in eating disorder symptoms [3, 36, 43]. It should be noted that patients who have undergone surgical procedures tend to be older [3], and thus improvement in eating disorder symptoms may also be secondary to the natural history of the illness, which in some cases does improve with age.

Eating disorders can serve as a barrier to gender affirming surgeries. Frequently, there are BMI requirements for gender-affirming surgeries, preventing those in larger bodies from receiving these interventions. Thresholds vary significantly among surgeons [50]. While it is increasingly understood that BMI is a problematic marker of health [51], there is concern that very high BMI may be associated with poorer surgical outcomes [52]. Thus, TGD patients may be encouraged to lose weight to become eligible for surgery [50, 53]. One study examining BMI at time of presentation for gender-affirming surgery found that 13% of patients were ineligible due to a BMI greater than 33 kg/m^2^ [53]. Of note, the BMI criteria particularly impacted ethnic minorities, who were more likely to have an elevated BMI. Patients were counseled that, to be eligible for gender-affirming surgery, they would need to lose weight. The study found that self-monitored weight loss was ineffective and no patients who were previously ineligible for surgery became eligible [53]. Indeed for most patients, weight remained stable or rose on follow-up [53]. Such high-stakes pressure to lose weight can exacerbate eating disordered behaviors [50, 54]. Notably, one study looking at post-operative complications in trans masculine individuals undergoing mastectomies did not find increased rates of complications for those with a BMI between 30 and 39.9 kg/m^2^ [55]. This suggests a need for future research in order to be able to accurately weigh the risks and benefits of gender affirming surgery for those in larger bodies.

For patients who are weight suppressed or otherwise unable to consistently nourish their bodies, there are no guidelines regarding the degree of weight restoration needed prior to pursuing gender-affirming surgeries. In these cases, it is important to carefully consider individual risks and benefits. Malnourishment places patients at risk of additional surgical complications. Adequate nourishment is important for wound healing and studies show increased mortality rates in surgical patients who are underweight [56, 57]. In addition, for those significantly underweight, anesthesia carries increased risk of hypoglycemia and leukocytopenia, with greatest risk for those with BMI less than 14 kg/m^2^ [58]. Both patients with anorexia and bulimia nervosa have been shown to have increased risk of cardiac complications under anesthesia [58, 59]. What constitutes sufficient stability from an eating disorder to optimize outcomes for gender affirming surgery is an area that requires additional research. Interestingly, a recent article by Murphy and colleagues examining outcomes in patients who had received facial gender affirming surgery found 5.4% of patients were underweight (BMI < 18.5) at the time of the procedure and that was not associated with increased risk of surgical complications [60]. It should also be noted that the literature reveals increased rates of eating disorders in cisgender patients requesting and receiving similar surgical procedures, such as breast reduction or augmentation, and was even shown in some cases to lead to a reduction in disordered eating behaviors [61, 62]. Thus, the presence of an eating disorder should not automatically prevent an individual from pursuing gender affirming surgery. Instead, patients may benefit from additional treatment and support prior to surgery to improve their eating disorder symptoms, increase their medical stability and optimize surgical outcomes. This is consistent with current WPATH SOC 8 guidelines which recommend mental health symptoms be addressed in order to enable an individual to engage appropriately in perioperative care [27].

## Additional medical considerations

### Bone density

Osteoporosis and osteopenia have long been known to be present in patients suffering from anorexia nervosa (AN). Increased evidence points to risk for those with avoidant restrictive food intake disorder (ARFID) and atypical anorexia as well [63–66]. While studies have been largely conducted with cisgender women, cisgender men with anorexia nervosa have also been found to be at increased risk of bone loss [65, 67]. Sex hormones serve a major role in bone formation and homeostasis [68]. However, research regarding the bone health of TGD patients with restrictive eating disorders is almost non-existent, limited to case reports [69].

Transgender women have lower bone density prior to starting GAHT when compared to cisgender men [70, 71]. In fact, the bone density of transgender women has been noted to be closer to that of cisgender women than to cisgender men [72]. Lower bone mineral density (BMD) in transgender women may be secondary to lower mean muscle mass and lower muscle strength [70], which would put transgender women at increased risk of bone loss with restrictive eating. In contrast, transgender men appear to have comparable bone mineral density when compared with cisgender women [73].

Gender affirming treatment can impact BMD. In adolescents, GnRH agonists may decrease BMD and may compound the bone density loss induced by restriction [74, 75]. Adolescence is a vulnerable time for BMD development as it represents the time of peak bone accrual. Given the additional detrimental effect of restriction on BMD, in addition to focusing on weight restoration, patients with restrictive eating disorders may benefit from relatively shorter duration of treatment with GnRH. Research in this area is lacking. For transgender woman, GAHT appear to cause no change or a slight increase in BMD [68]. Studies in transgender men tend to show stable, or sometimes slightly increasing BMD with testosterone therapy [68, 76]. Patients who have undergone orchiectomy or oophorectomy who take GAHT at low doses or inconsistently, may be at increased risk of low BMD [68].

DXA scans are routinely recommended for those with anorexia nervosa lasting longer than 6 months, particularly those recorded female at birth with greater than 6mo of amenorrhea [77]. While T-scores are calculated using a standardized data set of young women regardless of the sex of the patient, it is standard practice that Z-scores are matched by age, race and sex [78]. The International Society of Clinical Densitometry recommends that Z-scores be calculated based on the gender identity of the patient, rather than the sex recorded at birth [76], although clinical practice does not always follow these guidelines [79]. Given the complex hormonal milieu of many TGD patients, DXA scans can provide additional clinical information regarding bone health for TGD patients with restrictive eating disorders.

### Metabolic profile

For trans men using testosterone, studies have shown an increase in lean muscle mass and reduction in fat mass [80]. Testosterone has also been shown to lead to an increase in LDL cholesterol and decrease in HDL cholesterol, with mixed results on other lipid levels [80]. Testosterone does not appear to have a significant impact on insulin resistance [81]. Like with bone density, trans women are noted to have differences in body composition compared to cisgender men even prior to starting GAHT. Specifically, transgender women tend to have less lean mass and more fat mass at baseline [81]. Studies consistently show transgender women on GAHT have further decreases in lean mass and increases in fat mass, particularly with an increase in gynoid fat [81]. Whether there is an increase in insulin resistance with the administration of GAHT for transgender women is unclear [81].

Individuals with binge eating disorder have been shown to have increased risk of the components of metabolic syndrome, including central adiposity, insulin resistance, dyslipidemia and hypertension when compared with weight matched controls [82, 83]. Thus, TGD individuals with Binge Eating Disorder (BED) disorder may be at increased risk for development of metabolic syndrome and benefit from medical and psychiatric interventions.

### Weight

One component of eating disorder treatment is the establishment of a target weight. Typically, the establishment of a target weight is individualized and multifactorial, taking into consideration sex, age, previous weight trends, and growth charts. However, many aspects of the nutrition assessment include sex-specific values in their calculations [84–86]. Currently, the field lacks clinical practice standards for gender-affirming nutrition care [85]. GAHT contributes to increased BMI, particularly for transgender women, and thus may be considered in the calculation of target weight [80, 87, 88]. Clinicians are encouraged to collaborate with the patient and consider both cis male and cis female calculations to determine a medically stable, gender-affirming target weight [86]. Patients can benefit from target weight ranges that include both male and female values; target weight ranges may need to be adjusted up for those on GAHT [85].

### Healthcare bias

Similar to the experience individuals in larger bodies for whom all medical conditions are attributed to their weight, there may be a tendency to attribute the symptoms a transgender individual experiences to their gender identity or gender affirming medical interventions [89]. Fear of discrimination can lead to patients choosing not to disclose their gender identity. In a qualitative survey of the experience of transgender individuals in eating disorder treatment, Duffy and colleagues found that 40% of participants did not disclose they were transgender [90]. Given the importance of gender identity in the treatment of disordered eating this represents a significant missed opportunity to support and adequately care for these patients.

## Future research

The research on the intersection of TGD medical care and eating disorders is in its infancy. We are only just beginning to validate current eating disorder screening tools for the TGD population; while the EDE-Q has been validated in this population, many others currently in use have not and thus may miss aspects of TGD individuals’ symptom presentation [6, 91, 92]. Regarding treatment, there is limited evidence base on how to best support TGD patients in ED treatment, including with regards to their medical care. This includes how to optimize GAHT during eating disorder treatment and recovery. It is unknown, for example, what if any impact hormonal treatment has on the process of refeeding, including underlying physiological changes or rate of weight restoration. Similarly, the impact of GAHT on bone health in patients with restrictive eating disorders is unknown. It is reasonable to hypothesize that exogenous estrogen or testosterone could be protective against low BMD, whereas GnRH agonists may exacerbate bone loss. This could have clinical implications for how and when to start GAHT and further research is needed to inform clinical decision making. Regarding gender affirming surgical interventions, further studies are needed to determine how to best support patients with eating disorders who seek surgical interventions such that their eating disorder symptoms do not create unnecessary barriers to care. Additionally, longitudinal studies are needed to examine the impact of such surgeries on disordered eating symptoms. For the establishment of target weights, the field would benefit from the creation of gender inclusive calculations for weight and energy needs. Finally, it behooves us to continue to explore the intersectional nature of identity and work to understand and mitigate bias in the medical system and society at large in order to improve patient outcomes.

## Conclusions

Our understanding of the interplay between eating disorders and gender identify continues to evolve. As with other historically excluded communities, research in this area is severely lacking and the field would benefit from a concerted efforts to increase our understanding of the unique aspects of eating disorder treatment for TGD individuals. Despite this lack of data, it is important to follow best practices based on our current knowledge of both fields. As such, we recommend that eating disorder treatment facilities create environments that are inclusive for staff and patients alike, including use of correct names and pronouns and providing care that is gender inclusive and trauma informed. Patients of all ages can take steps in treatment to socially affirm their gender, and staff should be supportive of individual exploration. Patients who are already in the process of seeking out gender affirming medical interventions should be supported in doing so as part of their eating disorder treatment process, and providers can work with individuals to seek to reduce barriers to care created by their eating disorder. Those already on GAHT should continue with these medications throughout treatment and recovery from their eating disorder. Patients with restrictive eating disorders may benefit from more frequent monitoring of hormone levels due to the suppression of the HPA access secondary to malnourishment. Given the interaction between GAHT, restriction and bone health, monitoring BMD may be beneficial, particularly for adolescents who have been on GnRH agonists. Metabolic markers should be followed and treated appropriately. Target weights should take into consideration the patient’s gender identity as well as their hormonal status. TGD patients frequently cite lack of provider knowledge as a barrier to receiving adequate care [19]. All members of the care team can benefit from additional training in gender affirming care, as all members play an important role in supporting the patient’s treatment. Current evidence points to the necessity of providing gender affirming care within the context of eating disorder treatment.

## Data Availability

Data sharing is not applicable to this article as no datasets were generated or analyzed during the current study.
